# Supplementary material for: Fetal, neonatal, and infant outcomes associated with maternal Zika virus infection during pregnancy: A systematic review and meta-analysis
Source: PLoS One. 2021 Feb 19;16(2):e0246643. doi: 10.1371/journal.pone.0246643 (PMC7894820; doi:10.1371/journal.pone.0246643)
Supplement: S1 Table — (DOCX) [file pone.0246643.s002.docx]

| **Search Strategy – 15th November 2020** | | |
| --- | --- | --- |
| **DATABASE** | **STRATEGY TERMS USED** | **NUMBER OF STUDIES** |
| **Portal Regional da BVS**  **TERMS IN PORTUGUESE AND SPANISH** | tw: ( (“zika vírus” OR “vírus da zika” OR “vírus da febre zika” OR “vírus de zika” OR “vírus zika” OR “zikavirus” OR “zikv” OR “zikv”) AND (“microcefalia” OR “manifestações neurológicas” OR “manifestaciones neurológicas” OR “anormalidades congênitas” OR “anomalías congénitas” OR “microcefalias” OR “microcéfalo” OR “microencéfalo” OR “deficiências neurológicas” OR “déficits neurológicos” OR “sinais e sintomas neurológicos” OR “anomalia congênita” OR “anomalias congênitas” OR “anormalidade congênita” OR “malformações” OR “malformações congênitas” OR “deformidades” OR “defeitos congênitos”) AND (“epidemiologia” OR “epidemiología” OR “incidência” OR “incidencia” OR “prevalência” OR “prevalencia” OR “coorte” OR “cohorte” OR “ocorrência” OR “frequência” ) ) AND (instance:"regional") | 0 |
| **Portal Regional da BVS**  **TERMS IN ENGLISH** | tw: ( (“zika virus” OR “zika virus infection” OR “zikv” OR “virus, zika” OR “infection, zika virus” OR “virus infection, zika” OR “zikv infection” OR “fever, zika” OR “zika virus disease” OR “disease, zika virus” OR “virus disease, zika” OR “zika fever”) AND (“microcephaly” OR “congenital abnormalities” OR “nervous system diseases” OR “neurologic manifestations” OR “microcephalies” OR “congenital abnormality” OR “congenital defects” OR “birth defects” OR “cns disease” OR “cns diseases”) AND (“epidemiology” OR “prevalence” OR “incidence” OR “cohort” OR “frequency” OR “occurrence”) ) AND (instance:"regional") | 17 |
| **Pubmed** | ((("Zika Virus"[All Fields] OR "Zika Virus Infection"[MeSH Terms]) OR ("ZikV"[Title/Abstract] OR "ZikV Infection"[Title/Abstract] OR "Zika Virus Disease"[Title/Abstract] OR "Zika Fever"[Title/Abstract])) AND (("Microcephaly"[All Fields] OR "Congenital Abnormalities"[All Fields] OR "Nervous System Diseases"[All Fields] OR "Neurologic Manifestations"[MeSH Terms]) OR ("Microcephalies"[Title/Abstract] OR "Congenital Abnormality"[Title/Abstract] OR "Congenital Defects"[Title/Abstract] OR "Birth Defects"[Title/Abstract] OR "CNS Disease"[Title/Abstract] OR "CNS Diseases"[Title/Abstract]))) AND (("Epidemiology"[All Fields] OR "Prevalence"[All Fields] OR "Incidence"[MeSH Terms]) OR ("Cohort"[Title/Abstract] OR "Frequency"[Title/Abstract] OR "Occurrence"[Title/Abstract])) | 985 |
| **CAPES PORTAL**  **TERMS IN ENGLISH** | ("Zika Virus" OR "Zika Virus Infection" OR "ZikV" OR "Virus, Zika" OR "Infection, Zika Virus" OR "Virus Infection, Zika" OR "ZikV Infection" OR "Fever, Zika" OR "Zika Virus Disease" OR "Disease, Zika Virus" OR "Virus Disease, Zika" OR "Zika Fever") AND ("Microcephaly" OR "Congenital Abnormalities" OR "Nervous System Diseases" OR "Neurologic Manifestations" OR "Microcephalies" OR "Congenital Abnormality" OR "Congenital Defects" OR "Birth Defects" OR "CNS Disease" OR "CNS Diseases") AND ("Epidemiology" OR "Prevalence" OR "Incidence" OR "Cohort" OR "Frequency" OR "Occurrence") | 434 |
| **CAPES PORTAL**  **TERMS IN PORTUGUESE AND SPANISH** | (“zika vírus” OR “vírus da zika” OR “vírus da febre zika” OR “vírus de zika” OR “vírus zika” OR “zikavirus” OR “zikv” OR “zikv”) AND (“microcefalia” OR “manifestações neurológicas” OR “manifestaciones neurológicas” OR “anormalidades congênitas” OR “anomalías congénitas” OR “microcefalias” OR “microcéfalo” OR “microencéfalo” OR “deficiências neurológicas” OR “déficits neurológicos” OR “sinais e sintomas neurológicos” OR “anomalia congênita” OR “anomalias congênitas” OR “anormalidade congênita” OR “malformações” OR “malformações congênitas” OR “deformidades” OR “defeitos congênitos”) AND (“epidemiologia” OR “epidemiología” OR “incidência” OR “incidencia” OR “prevalência” OR “prevalencia” OR “coorte” OR “cohorte” OR “ocorrência” OR “frequência” ) | 22 |
| **SCOPUS** | ( TITLE-ABS-KEY ( "Zika Virus"  OR  "Zika Virus Infection"  OR  "ZikV"  OR  "Virus, Zika" )  OR  TITLE-ABS-KEY ( "Infection, Zika Virus"  OR  "Virus Infection, Zika"  OR  "ZikV Infection"  OR  "Fever, Zika" )  OR  TITLE-ABS-KEY ( "Zika Virus Disease"  OR  "Disease, Zika Virus"  OR  "Virus Disease, Zika"  OR  "Zika Fever" )  AND  TITLE-ABS-KEY ( "Microcephaly"  OR  "Congenital Abnormalities"  OR  "Nervous System Diseases"  OR  "Neurologic Manifestations"  OR  "Microcephalies" )  OR  TITLE-ABS-KEY ( "Congenital Abnormality"  OR  "Congenital Defects"  OR  "Birth Defects"  OR  "CNS Disease"  OR  "CNS Diseases" )  AND  TITLE-ABS-KEY ( "Epidemiology"  OR  "Prevalence"  OR  "Incidence"  OR  "Cohort"  OR  "Frequency"  OR  "Occurrence" ) ) | 515 |
| **WEB OF SCIENCE** | ALL FIELDS:(“Zika Virus” OR “Zika Virus Infection” OR “ZikV” OR “Virus, Zika” OR “Infection, Zika Virus” OR “Virus Infection, Zika” OR “ZikV Infection” OR “Fever, Zika” OR “Zika Virus Disease” OR “Disease, Zika Virus” OR “Virus Disease, Zika” OR “Zika Fever”)*AND* ALL FIELDS: (“Microcephaly” OR “Congenital Abnormalities” OR “Nervous System Diseases” OR “Neurologic Manifestations” OR “Microcephalies” OR “Congenital Abnormality” OR “Congenital Defects” OR “Birth Defects” OR “CNS Disease” OR “CNS Diseases”) *AND* ALL FIELDS: (“Epidemiology” OR “Prevalence” OR “Incidence” OR “Cohort” OR “Frequency” OR “Occurrence”)  Timespan: All years. Indexes: SCI-EXPANDED, SSCI, A&HCI, CPCI-S, CPCI-SSH, ESCI. | 345 |
| **Cochrane Library** | “Zika Virus” OR “Zika Virus Infection” OR “ZikV” OR “Virus, Zika” OR “Infection, Zika Virus” OR “Virus Infection, Zika” OR “ZikV Infection” OR “Fever, Zika” OR “Zika Virus Disease” OR “Disease, Zika Virus” OR “Virus Disease, Zika” OR “Zika Fever” in Title Abstract Keyword AND “Microcephaly” OR “Congenital Abnormalities” OR “Nervous System Diseases” OR “Neurologic Manifestations” OR “Microcephalies” OR “Congenital Abnormality” OR “Congenital Defects” OR “Birth Defects” OR “CNS Disease” OR “CNS Diseases” in Title Abstract Keyword AND “Epidemiology” OR “Prevalence” OR “Incidence” OR “Cohort” OR “Frequency” OR “Occurrence” in Title Abstract Keyword - (Word variations have been searched)' | 3 |
| **Scielo**  **TERMS IN PORTUGUESE AND SPANISH** | (“zika vírus” OR “vírus da zika” OR “vírus da febre zika” OR “vírus de zika” OR “vírus zika” OR “zikavirus” OR “zikv” OR “zikv”) AND (“microcefalia” OR “manifestações neurológicas” OR “manifestaciones neurológicas” OR “anormalidades congênitas” OR “anomalías congénitas” OR “microcefalias” OR “microcéfalo” OR “microencéfalo” OR “deficiências neurológicas” OR “déficits neurológicos” OR “sinais e sintomas neurológicos” OR “anomalia congênita” OR “anomalias congênitas” OR “anormalidade congênita” OR “malformações” OR “malformações congênitas” OR “deformidades” OR “defeitos congênitos”) AND (“epidemiologia” OR “epidemiología” OR “incidência” OR “incidencia” OR “prevalência” OR “prevalencia” OR “coorte” OR “cohorte” OR “ocorrência” OR “frequência” ) | 0 |
| **Scielo**  **TERMS IN ENGLISH** | (“Zika Virus” OR “Zika Virus Infection” OR “ZikV” OR “Virus, Zika” OR “Infection, Zika Virus” OR “Virus Infection, Zika” OR “ZikV Infection” OR “Fever, Zika” OR “Zika Virus Disease” OR “Disease, Zika Virus” OR “Virus Disease, Zika” OR “Zika Fever”) AND (“Microcephaly” OR “Congenital Abnormalities” OR “Nervous System Diseases” OR “Neurologic Manifestations” OR “Microcephalies” OR “Congenital Abnormality” OR “Congenital Defects” OR “Birth Defects” OR “CNS Disease” OR “CNS Diseases”) AND (“Epidemiology” OR “Prevalence” OR “Incidence” OR “Cohort” OR “Frequency” OR “Occurrence”) | 0 |
| **LILACS**  **TERMS IN PORTUGUESE AND SPANISH** | (“zika vírus” OR “vírus da zika” OR “vírus da febre zika” OR “vírus de zika” OR “vírus zika” OR “zikavirus” OR “zikv” OR “zikv”) AND (“microcefalia” OR “manifestações neurológicas” OR “manifestaciones neurológicas” OR “anormalidades congênitas” OR “anomalías congénitas” OR “microcefalias” OR “microcéfalo” OR “microencéfalo” OR “deficiências neurológicas” OR “déficits neurológicos” OR “sinais e sintomas neurológicos” OR “anomalia congênita” OR “anomalias congênitas” OR “anormalidade congênita” OR “malformações” OR “malformações congênitas” OR “deformidades” OR “defeitos congênitos”) AND (“epidemiologia” OR “epidemiología” OR “incidência” OR “incidencia” OR “prevalência” OR “prevalencia” OR “coorte” OR “cohorte” OR “ocorrência” OR “frequência” ) | 0 |
| **LILACS**  **TERMS IN ENGLISH** | (“Zika Virus” OR “Zika Virus Infection” OR “ZikV” OR “Virus, Zika” OR “Infection, Zika Virus” OR “Virus Infection, Zika” OR “ZikV Infection” OR “Fever, Zika” OR “Zika Virus Disease” OR “Disease, Zika Virus” OR “Virus Disease, Zika” OR “Zika Fever”) AND (“Microcephaly” OR “Congenital Abnormalities” OR “Nervous System Diseases” OR “Neurologic Manifestations” OR “Microcephalies” OR “Congenital Abnormality” OR “Congenital Defects” OR “Birth Defects” OR “CNS Disease” OR “CNS Diseases”) AND (“Epidemiology” OR “Prevalence” OR “Incidence” OR “Cohort” OR “Frequency” OR “Occurrence”) | 0 |
| **Google Scholar** | (“Zika Virus” OR “Zika Virus Infection” OR “ZikV” OR “Virus, Zika” OR “Infection, Zika Virus” OR “Virus Infection, Zika” OR “ZikV Infection” OR “Fever, Zika” OR “Zika Virus Disease” OR “Disease, Zika Virus” OR “Virus Disease, Zika” OR “Zika Fever”) AND (“Microcephaly” OR “Congenital Abnormalities” OR “Nervous System Diseases” OR “Neurologic Manifestations” OR “Microcephalies” OR “Congenital Abnormality” OR “Congenital Defects” OR “Birth Defects” OR “CNS Disease” OR “CNS Diseases”) AND (“Epidemiology” OR “Prevalence” OR “Incidence” OR “Cohort” OR “Frequency” OR “Occurrence”) | 1203 |
| **CINAHL** | ( “Zika Virus” OR “Zika Virus Infection” OR “ZikV” OR “Virus, Zika” OR “Infection, Zika Virus” OR “Virus Infection, Zika” OR “ZikV Infection” OR “Fever, Zika” OR “Zika Virus Disease” OR “Disease, Zika Virus” OR “Virus Disease, Zika” OR “Zika Fever” ) AND TX ( “Microcephaly” OR “Congenital Abnormalities” OR “Nervous System Diseases” OR “Neurologic Manifestations” OR “Microcephalies” OR “Congenital Abnormality” OR “Congenital Defects” OR “Birth Defects” OR “CNS Disease” OR “CNS Diseases” ) AND TX ( “Epidemiology” OR “Prevalence” OR “Incidence” OR “Cohort” OR “Frequency” OR “Occurrence” ) | 390 |
