# Supplementary material for: Fetal, neonatal, and infant outcomes associated with maternal Zika virus infection during pregnancy: A systematic review and meta-analysis
Source: PLoS One. 2021 Feb 19;16(2):e0246643. doi: 10.1371/journal.pone.0246643 (PMC7894820; doi:10.1371/journal.pone.0246643)
Supplement: S2 Table — (DOCX) [file pone.0246643.s003.docx]

**S2 Table: Critical appraisal checklist for studies reporting prevalence data.**

|  | Quality Assesment Criteria | |
| --- | --- | --- |
| 1 | Were study participants sampled in a appropriate way? | Selection bias |
| 2 | Was a clear, standard definition used for maternal ZIKV infection? | Measurement bias |
| 3 | Was infection measured reliably using trained/educated data collectors, appropriate/reliable diagnostic procedures, or reliable forms of retrospective data (clinical records meeting standard definitions)? | Measurement bias |
| 4 | Was the condition measured in a standard, reliable way for all participants? | Measurement bias |
| 5 | Were study subjects and setting described in suficiente detail to determine whether results are comparable with other studies? | Poor characterisation of study population |

Adapted from Munn et al. 2015. DOI: 10.1097/XEB.0000000000000054.
