# Supplementary material for: Fetal, neonatal, and infant outcomes associated with maternal Zika virus infection during pregnancy: A systematic review and meta-analysis
Source: PLoS One. 2021 Feb 19;16(2):e0246643. doi: 10.1371/journal.pone.0246643 (PMC7894820; doi:10.1371/journal.pone.0246643)
Supplement: S3 Table — (DOCX) [file pone.0246643.s004.docx]

**S3 Table: Quality of 21 included cohort studies**

| **Yes** |  |
| --- | --- |
| **Unclear** |  |
| **No** |  |

| **Study** | **Sampling** | **Definition ZIKV infection** | **Data collection** | **Definition outcome** | **Sufficient detail** |
| --- | --- | --- | --- | --- | --- |
| Adams et al. 2016 |  |  |  |  |  |
| Adhikari et al. 2017 |  |  |  |  |  |
| Aspilcueta-Gho et al. 2016 |  |  |  |  |  |
| Brasil et al. 2016 |  |  |  |  |  |
| Hall et al. 2017 |  |  |  |  |  |
| Hoen et al. 2018 |  |  |  |  |  |
| Honein et al. 2017 |  |  |  |  |  |
| João et al. 2018 |  |  |  |  |  |
| Mendez et al. 2017 |  |  |  |  |  |
| Mulkey et al. 2018 |  |  |  |  |  |
| Pomar et al. 2017 |  |  |  |  |  |
| Reynolds et al. 2017 |  |  |  |  |  |
| Rice et al. 2018 |  |  |  |  |  |
| Rodriguez-Morales et al. 2018 |  |  |  |  |  |
| Shapiro-Mendonza et al. 2017 |  |  |  |  |  |
| de Sousa et al. 2020 |  |  |  |  |  |
| Clemente et al. 2020 |  |  |  |  |  |
| Ocampo Canas et al. 2020 |  |  |  |  |  |
| Ospina et al. 2020 |  |  |  |  |  |
| Brasil et al. 2020 |  |  |  |  |  |
| Coutinho et al. 2020 |  |  |  |  |  |
