# Supplementary material for: Fetal, neonatal, and infant outcomes associated with maternal Zika virus infection during pregnancy: A systematic review and meta-analysis
Source: PLoS One. 2021 Feb 19;16(2):e0246643. doi: 10.1371/journal.pone.0246643 (PMC7894820; doi:10.1371/journal.pone.0246643)
Supplement: Attachment — Submitted filename: Response to Reviewer.docx [file pone.0246643.s006.docx]

Frank T. Spradley

Academic Editor

PLOS ONE

Dear Mr Spradley

Once again, we would like to express our full appreciation for allowing us to revise our manuscript entitled " Fetal, neonatal, and infant outcomes associated with maternal Zika virus infection during pregnancy: a systematic review and meta-analysis ". We believe that the inclusion o fone more cohort, previously not found in our research, has helped to improve the manuscript ultimately. All the changes in the paper are highlighted in "red" in the revised version (Revised Manuscript Track Changes).

I remain at your disposal to clarify any pending point.

Yours sincerely,

Marlos Melo Martins

Universidade Federal do Rio de Janeiro

Rua das Laranjeiras 180, Rio de Janeiro, RJ, Brazil

Zip Code: 22240-000

**Dear Reviewer:**

Thank you very much for the new comments. Your insightful suggestion of including a new cohort immensely helped to improve the manuscript ultimately. All the changes in the paper are highlighted in "red" in the revised version (Revised Manuscript Track Changes). We also tried to improve our english in the manuscript.

I remain at your disposal to clarify any pending point.

Yours sincerely,

Marlos Melo Martins

Universidade Federal do Rio de Janeiro

Rua das Laranjeiras 180, Rio de Janeiro, RJ, Brazil

Zip Code: 22240-000
